# U-shaped association between TC/HDL-C ratio and osteoporosis risk in older adults

**DOI:** 10.1038/s41598-025-89537-5

**Published:** 2025-02-08

**Authors:** Chuanwei Zhao, Xiaochun Zhang, Xu Zhang, Bo Zhao, Yane Yang, Mu Lin, Wenli Qiao, Zeyao Hu, Haijie Yang

**Affiliations:** Department of Cardiology, The Second People’s Hospital of Baoshan, No.13, Zhengyang (S) Rd., Longyang Dist., Baoshan, Yunnan China

**Keywords:** Osteoporosis, TC/HDL-C ratio, Lipid metabolism, Elderly population, NHANES, Diagnostic markers, Predictive markers, Endocrine system and metabolic diseases, Dyslipidaemias, Metabolic bone disease

## Abstract

This study aimed to evaluate the association between the total cholesterol to high-density lipoprotein cholesterol (TC/HDL-C) ratio and the risk of osteoporosis in older adults. A cross-sectional analysis was conducted using data from 7,465 individuals aged 60 and above from the 2005–2020 National Health and Nutrition Examination Survey (NHANES). The TC/HDL-C ratio was calculated and divided into quartiles. Osteoporosis was defined by self-reported physician diagnosis or dual-energy x-ray absorptiometry (DXA) measurements. Multivariable logistic regression models assessed the association between the TC/HDL-C ratio and osteoporosis prevalence. Restricted cubic splines were used to explore non-linear associations and threshold effects. Among the participants, 1,608 had osteoporosis. A higher TC/HDL-C ratio was inversely associated with osteoporosis (OR: 0.92, 95% CI: 0.86–0.99, *p* = 0.02). The analysis revealed a U-shaped association (*p* for nonlinearity = 0.005) with a threshold at 4.66. Below this threshold, the TC/HDL-C ratio was negatively associated with osteoporosis (OR: 0.83, 95% CI: 0.72–0.94, *p* = 0.010), while no significant association was observed above the threshold. In sex-specific analyses, a U-shaped relationship was found in women, with a threshold at 4.35 (*p* for nonlinearity = 0.0016), but no significant association was found in men. The TC/HDL-C ratio demonstrates a U-shaped association with osteoporosis risk in older adults, particularly in women. Further longitudinal studies are required to validate these findings and determine optimal lipid management strategies for osteoporosis prevention.

**Supplementary Information:**

The online version contains supplementary material available at 10.1038/s41598-025-89537-5.

## Introduction

Osteoporosis is a common condition affecting millions of individuals worldwide, especially the elderly. Characterized by decreased bone mineral density (BMD) and increased risk of fractures, it is a major public health issue that leads to significant morbidity and healthcare costs^1^. The increasing aging population has heightened the importance of understanding risk factors for osteoporosis, as it is a leading cause of fractures, disability, and diminished quality of life in older adults^2,3^. Despite well-established risk factors such as age, gender, and genetics, there is growing interest in the potential role of metabolic factors, including lipid profiles, in influencing bone health.

Lipid metabolism has been implicated in various physiological processes, including bone remodeling^4^. Cholesterol, particularly the balance between total cholesterol (TC) and high-density lipoprotein cholesterol (HDL-C), may influence the activities of osteoblasts (bone-forming cells) and osteoclasts (bone-resorbing cells). HDL-C is known for its anti-inflammatory and antioxidative properties, which could protect bone cells from oxidative stress and promote bone formation^5,6^. Conversely, higher TC levels, commonly associated with cardiovascular risk, may lead to increased inflammation and oxidative stress, potentially contributing to bone loss^7^. The TC/HDL-C ratio, as a widely used lipid metabolism marker, reflects the overall balance of cholesterol^8^. It integrates the protective effects of HDL-C and the potential adverse impacts of TC, making it a valuable metric in studies of cardiovascular and metabolic diseases.

Previous research has explored the link between lipid profiles and bone health, though the findings have been inconsistent. Some studies suggest that dyslipidemia, including elevated TC/HDL-C ratios, is associated with lower BMD and an increased risk of osteoporosis^9,10^. For instance, studies in postmenopausal women have shown a negative correlation between HDL-C levels and osteoporosis risk^11^. However, other research has found no significant relationship between cholesterol levels and BMD, or even a positive association^12–14^. These conflicting results may be due to differences in study populations, variations in lipid measurements, or the complex interplay between lipid metabolism and bone health across different demographic groups.

Given the mixed evidence, further investigation is necessary to clarify the relationship between lipid profiles and osteoporosis, particularly in older populations who are most at risk. The TC/HDL-C ratio, a widely used marker for cardiovascular risk, could also serve as a potential biomarker for bone health^15^. Moreover, sex-specific differences in lipid metabolism, influenced by hormones such as estrogen, suggest that the impact of lipid levels on bone health may vary between men and women^16,17^. Understanding these differences is crucial for developing targeted strategies to mitigate osteoporosis risk. This study aims to investigate the association between the TC/HDL-C ratio and osteoporosis in older adults using data from the National Health and Nutrition Examination Survey (NHANES) from 2005 to 2020. We also explore whether this relationship differs by sex, age, and other demographic factors. By providing further insights into the role of lipid metabolism in bone health, this research may help identify new strategies for osteoporosis prevention and management.

## Materials and methods

### Study design and participants

This study utilized publicly available data from the NHANES, conducted by the Centers for Disease Control and Prevention (CDC). NHANES aims to assess the health and nutritional status of the U.S. population. Ethical approval for NHANES was granted by the Institutional Review Board of the National Center for Health Statistics (NCHS), and all participants provided written informed consent prior to enrollment^18^. Data collected includes demographic information, questionnaire responses, medical examination results, and laboratory measurements.

**Fig. 1:**
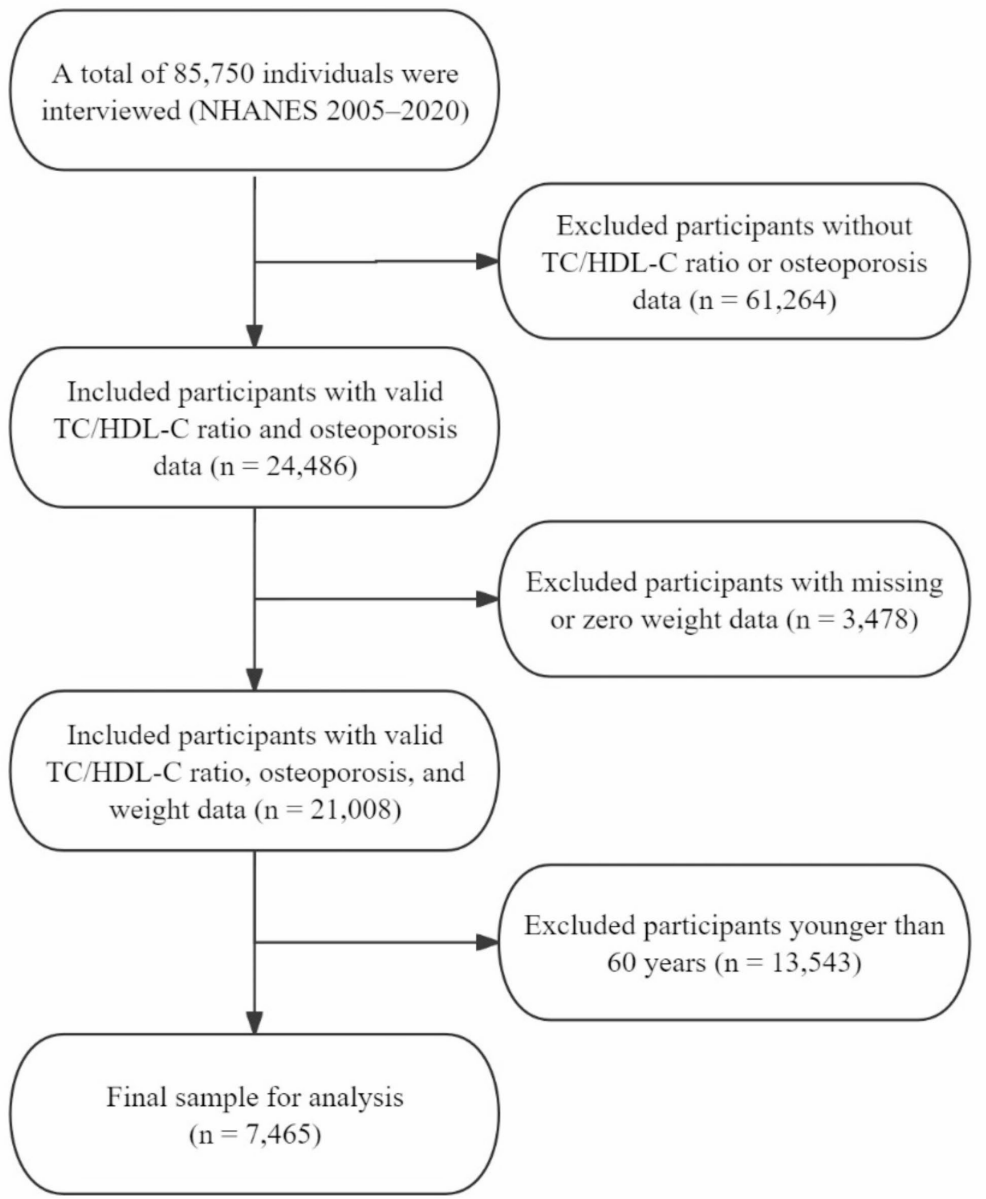
Flowchart of participant selection for the analysis.

A total of 85,750 participants were enrolled in NHANES from 2005 to 2020. After excluding individuals without records for TC, HDL-C, osteoporosis diagnosis, weight data, or those under the age of 60, the remaining sample was used for analysis (Fig. 1).

### Lipid measurement

Lipid measurements were conducted following standardized protocols from the CDC. Serum TC and HDL-C levels were measured enzymatically using a Hitachi 704 Analyzer (Boehringer Mannheim Diagnostics, Indianapolis, IN, USA)^19^. HDL-C was determined by direct immunoassay or precipitation methods^20^. LDL-C was calculated using the Friedewald formula [LDL-C = TC − HDL-C − (TG/5)] for participants with triglyceride (TG) levels ≤ 400 mg/dL^21^. According to NHANES guidelines, variations in measurement instruments were not considered. Participants were categorized into quartiles (Q1, Q2, Q3, Q4) based on their TC/HDL-C ratio.

### Osteoporosis assessment

Osteoporosis was defined according to the World Health Organization (WHO) criteria as either a self-reported physician diagnosis or, in the absence of such a history, a dual-energy x-ray absorptiometry (DXA) measurement showing a T-score of ≤ -2.5 at the femoral neck or lumbar spine (L1-3)^1,22^.

### Demographic characteristics and other covariates

Race/ethnicity was categorized as Mexican American, Non-Hispanic Black, Non-Hispanic White, or other race, based on survey design. Educational attainment was classified as below high school, high school graduate or GED, and some college or above. Marital status was grouped into married or living with a partner, never married, and widowed, divorced, or separated. The poverty income ratio (PIR), an index reflecting income relative to the federal poverty threshold, adjusted for inflation and family size, was used to assess economic status. Smoking status was categorized as never smoker, former smoker, and current smoker. Physical activity levels were assessed using weekly Metabolic Equivalent Tasks (METs) for activities such as walking, cycling, exercising, and engaging in leisure. Alcohol use was divided into active and non-active users. A history of atherosclerotic cardiovascular disease (ASCVD) included self-reported angina, congestive heart failure, coronary heart disease, myocardial infarction, or stroke.

Weight, height, and waist circumference were measured using standard protocols at mobile examination centers. Body mass index (BMI) was calculated as weight divided by height squared. Clinical measures from NHANES laboratory tests included total 25-hydroxyvitamin D, fasting plasma glucose, albumin, serum creatinine, uric acid, phosphorus, calcium, triglycerides, total cholesterol, HDL-C, low-density lipoprotein cholesterol (LDL-C), lymphocyte count, monocyte count, and hemoglobin. Estimated glomerular filtration rate (eGFR) was calculated using the chronic kidney disease epidemiology collaboration (CKD-EPI) equation^23^.

### Statistical analysis

The NHANES dataset, a multistage, stratified, probability-based survey, was analyzed using recommended sampling weights to account for unequal sampling probabilities and nonresponse^18^. Detailed descriptions of the sampling design and weighting are available on the NHANES website (https://wwwn.cdc.gov/nchs/nhanes/analyticguidelines.aspx). TC/HDL-C was categorized into quartiles for analysis: Q1 (TC/HDL-C < 2.90, reference), Q2 (2.90 ≤ TC/HDL-C < 3.56), Q3 (3.56 ≤ TC/HDL-C < 4.48), and Q4 (TC/HDL-C ≥ 4.48).

Baseline characteristics were compared between groups based on the presence of osteoporosis. Continuous variables were presented as mean ± standard deviation (SD) and compared using t-tests, while categorical variables were presented as counts (weighted percentages) and analyzed using Pearson’s chi-square test. Weighted univariate and multivariate logistic regression models were used to evaluate the associations of TC, HDL-C, and the TC/HDL-C ratio with osteoporosis, reporting odds ratios (ORs) and 95% confidence intervals (CIs). To enhance effect size interpretation, TC and HDL-C were scaled by dividing by 10 (denoted as TC/10 and HDL-C/10, respectively). To further evaluate the robustness of the findings, we conducted a sensitivity analysis by excluding individuals taking lipid-lowering medications and repeated the logistic regression analyses. Restricted cubic spline (RCS) curves were employed to assess potential non-linear associations between TC/HDL-C and osteoporosis. The number of knots in the RCS model was determined using the Akaike Information Criterion (AIC), where lower AIC values indicate a better-fitting model. Based on this criterion, three knots were selected, positioned at the 10th, 50th, and 90th percentiles of the TC/HDL-C distribution by default. Three models were constructed: Model 1 (unadjusted), Model 2 (adjusted for age, sex, and race), and Model 3 (adjusted for additional covariates, including BMI, education level, marital status, poverty income ratio [PIR], smoking, alcohol use, physical activity, hypertension, diabetes mellitus, atherosclerotic cardiovascular disease, cancer, steroid use, estrogen use, serum uric acid, serum albumin, eGFR, serum calcium, serum phosphorus, and total 25-hydroxyvitamin D). The goodness-of-fit of Model 3 was assessed using the Hosmer-Lemeshow test, and a *p*-value > 0.05 was considered indicative of an acceptable model fit.

Covariates were selected based on established associations with osteoporosis, and multiple imputation was applied for missing data^24^, yielding consistent results with complete-case analyses. Interaction and stratified analyses were conducted by sex, age, race, BMI, eGFR, serum calcium, serum phosphorus, and total 25-hydroxyvitamin D.

All statistical analyses were performed using R (v4.3.2). A two-sided *p*-value < 0.05 was considered statistically significant.

## Results

### Baseline characteristics

The study included 7,465 participants, with 1,608 in the osteoporosis group and 5,857 in the non-osteoporosis group (Table 1). Participants with osteoporosis were generally older and more likely to be female compared to those without osteoporosis. A greater proportion of participants with osteoporosis were widowed, divorced, or separated, had lower educational attainment, and had a lower poverty-to-income ratio. In terms of physical characteristics, participants with osteoporosis had a lower body mass index, smaller waist circumference, and reported lower levels of physical activity. The proportion of never-smokers and non-active alcohol users was higher among participants with osteoporosis compared to those without. Regarding clinical measurements, participants with osteoporosis had lower eGFR, lower serum albumin levels, and higher serum phosphorus and calcium levels compared to those without osteoporosis. Additionally, participants with osteoporosis had higher HDL-C levels and lower TC/HDL-C ratios. The use of steroids was also more frequent among participants with osteoporosis.

**Table 1:** Baseline characteristics of study participants.

Characteristic	Overall (*N* = 7465)	Non-osteoporosis (*N* = 5857)	Osteoporosis (*N* = 1608)	*p*-value
Age, years	69.70 ± 0.12	68.86 ± 0.12	72.49 ± 0.25	< 0.0001
Sex (n, %)				< 0.0001
Female	3706(54.66)	2377(45.29)	1329(85.92)	
Male	3759(45.34)	3480(54.71)	279(14.08)	
Race (n, %)				< 0.0001
Mexican American	971(4.28)	769(4.31)	202(4.18)	
Non-Hispanic Black	1406(7.82)	1239(8.76)	167(4.67)	
Non-Hispanic White	3951(79.42)	2982(78.67)	969(81.93)	
Other race	1137(8.48)	867(8.26)	270(9.22)	
Education level (n, %)				< 0.001
Below high school	2254(19.10)	1736(18.13)	518(22.32)	
High school graduate or GED	1829(26.37)	1400(25.59)	429(28.97)	
Some college or above	3382(54.53)	2721(56.27)	661(48.72)	
Marital status (n, %)				< 0.0001
Married or living with a partner	4434(64.25)	3682(68.16)	752(51.19)	
Never married	342(3.55)	271(3.59)	71(3.41)	
Widowed, divorced, or separated	2689(32.20)	1904(28.25)	785(45.40)	
Poverty-to-income ratio	3.07 ± 0.04	3.17 ± 0.04	2.72 ± 0.06	< 0.0001
Body mass index, kg/m²	28.57 ± 0.10	28.91 ± 0.09	27.42 ± 0.24	< 0.0001
Waist circumference, cm	101.30 ± 0.27	102.67 ± 0.24	96.71 ± 0.71	< 0.0001
Physical activity total METs/week	2766.96 ± 82.89	2962.47 ± 91.88	2114.89 ± 107.24	< 0.0001
Smoking status (n, %)				< 0.0001
Never	3640(49.60)	2733(47.93)	907(55.20)	
Former	2876(39.23)	2357(40.97)	519(33.43)	
Now	949(11.16)	767(11.10)	182(11.37)	
Alcohol use (n, %)				< 0.0001
Active alcohol user	4422(65.61)	3612(68.26)	810(56.77)	
Non-active alcohol user	3043(34.39)	2245(31.74)	798(43.23)	
Hypertension (n, %)				0.080
No	2407(34.82)	1938(35.63)	469(32.13)	
Yes	5058(65.18)	3919(64.37)	1139(67.87)	
Diabetes mellitus (n, %)				0.002
No	5107(73.87)	3955(72.76)	1152(77.58)	
Yes	2358(26.13)	1902(27.24)	456(22.42)	
ASCVD (n, %)				0.08
No	5839(79.03)	4626(79.88)	1213(76.18)	
Yes	1626(20.97)	1231(20.12)	395(23.82)	
Cancer (n, %)				0.002
No	5972(76.23)	4741(77.38)	1231(72.40)	
Yes	1493(23.77)	1116(22.62)	377(27.60)	
Total 25-hydroxyvitamin D, nmol/L	77.51 ± 0.73	75.76 ± 0.73	83.36 ± 1.42	< 0.0001
eGFR, mL/min/1.73 m²	72.97 ± 0.25	73.96 ± 0.28	69.65 ± 0.56	< 0.0001
Fasting plasma glucose, mg/dL	115.79 ± 0.57	116.87 ± 0.55	112.17 ± 1.14	< 0.0001
Albumin, g/dL	41.49 ± 0.07	41.66 ± 0.06	40.90 ± 0.13	< 0.0001
Serum creatinine, mg/dL	0.98 ± 0.00	0.99 ± 0.01	0.95 ± 0.01	< 0.001
Uric acid, mg/dL	5.63 ± 0.02	5.74 ± 0.03	5.26 ± 0.05	< 0.0001
Phosphorus, mmol/L	1.21 ± 0.00	1.19 ± 0.00	1.24 ± 0.01	< 0.0001
Calcium, mmol/L	2.32 ± 0.00	2.32 ± 0.00	2.34 ± 0.00	< 0.0001
Triglycerides, mg/dL	215.77 ± 3.35	215.43 ± 3.56	216.88 ± 7.22	0.850
Total cholesterol, mg/dL	194.69 ± 0.85	193.90 ± 0.91	197.33 ± 1.64	0.050
HDL-C, mg/dL	55.43 ± 0.32	54.00 ± 0.34	60.20 ± 0.64	< 0.0001
LDL-C, mg/dL	96.65 ± 0.83	97.41 ± 0.91	94.15 ± 1.54	0.050
Lymphocyte count, 1000 cells/µL	2.02 ± 0.02	2.02 ± 0.03	2.01 ± 0.03	0.800
Monocyte count, 1000 cells/µL	0.59 ± 0.00	0.59 ± 0.00	0.59 ± 0.01	0.990
Hemoglobin, g/dL	14.06 ± 0.04	14.21 ± 0.04	13.55 ± 0.05	< 0.0001
TC/HDL-C	3.76 ± 0.02	3.83 ± 0.02	3.52 ± 0.04	< 0.0001
Steroid use (n, %)				0.003
No	7171(95.77)	5650(96.40)	1521(93.64)	
Yes	294(4.23)	207(3.60)	87(6.36)	
Estrogen use (n, %)				0.930
No	7280(96.44)	5720(96.45)	1560(96.39)	
Yes	185(3.56)	137(3.55)	48(3.61)	
Lipid-lowering medication use (n, %)				0.600
No	4220(55.09)	3336(54.86)	884(55.86)	
Yes	3245(44.91)	2521(45.14)	724(44.14)	

Continuous variables are presented as mean ± standard deviation (SD).

Categorical variables are presented as number (percentage).

*GED* General educational development, *MET* Metabolic equivalent of task, *ASCVD* Atherosclerotic cardiovascular disease, *eGFR* Estimated glomerular filtration rate, *HDL-C* High-density lipoprotein cholesterol, *LDL-C* Low-density lipoprotein cholesterol, *TC* Total cholesterol.

### Association between TC/HDL-C ratio and osteoporosis in elderly individuals: a weighted logistic regression analysis

A weighted logistic regression analysis was performed to assess the relationship between the TC/HDL-C ratio and osteoporosis prevalence in elderly individuals (Table 2). In the unadjusted model (Model 1), a significant inverse association was observed between the TC/HDL-C ratio and osteoporosis (OR: 0.80, 95% CI: 0.75–0.86, *p* < 0.0001). This association remained significant, though attenuated, after adjusting for age, sex, and race in Model 2 (OR: 0.90, 95% CI: 0.84–0.96, *p* = 0.003), and after adjusting for all covariates in Model 3 (OR: 0.92, 95% CI: 0.86–0.99, *p* = 0.02). The Hosmer-Lemeshow test (*χ*² = 6.273, *p* = 0.617) indicated excellent goodness-of-fit for Model 3. Additionally, after adjusting for all covariates, each 10 mg/dL increase in TC was associated with a 2% reduction in the odds of osteoporosis (OR: 0.98, 95% CI: 0.95–1.00), while each 10 mg/dL increase in HDL-C was associated with a 5% increase in the odds of osteoporosis (OR: 1.05, 95% CI: 1.00–1.11, *p* = 0.07), though the latter did not reach statistical significance. When categorized into quartiles, higher TC/HDL-C ratios (Q2, Q3, and Q4) were associated with significantly lower odds of osteoporosis compared with the reference quartile (Q1). In the fully adjusted model (Model 3), the ORs for Q2, Q3, and Q4 were 0.77 (95% CI: 0.63–0.95, *p* = 0.02), 0.80 (95% CI: 0.65–1.00, *p* = 0.05), and 0.80 (95% CI: 0.64–0.99, *p* = 0.04), respectively. A significant trend across quartiles was evident in all models (*p* for trend < 0.05).

**Table 2:** Weighted logistic regression analysis of TC, HDL-C, and TC/HDL-C ratio in relation to osteoporosis prevalence in elderly individuals.

Characteristic	Model 1		Model 2		Model 3	
	OR (95%CI)	*p*	OR (95%CI)	*p*	OR (95%CI)	*p*
TC/10	1.02(1.00,1.04)	0.05	0.97(0.95,1.00)	0.02	0.98(0.95,1.00)	0.03
HDL-C/10	1.24(1.18,1.30)	< 0.0001	1.07(1.02,1.13)	0.01	1.05(1.00,1.11)	0.07
TC/HDL-C	0.80(0.75,0.86)	< 0.0001	0.90(0.84,0.96)	0.003	0.92(0.86,0.99)	0.02
Q1	Reference	–	Reference	–	Reference	–
Q2	0.67(0.55,0.82)	< 0.001	0.71(0.58,0.87)	0.001	0.77(0.63,0.95)	0.02
Q3	0.54(0.45,0.65)	< 0.0001	0.72(0.59,0.88)	0.001	0.80(0.65,1.00)	0.05
Q4	0.50(0.41,0.60)	< 0.0001	0.72(0.58,0.90)	0.004	0.80(0.64,0.99)	0.04
*p* for trend		< 0.0001		0.003		0.048

Model 1: Unadjusted.

Model 2: Adjusted for age, sex, and race.

Model 3: Adjusted for age, sex, race, body mass index (BMI), education level, marital status, ratio of family income to poverty (PIR), smoking status, alcohol use, physical activity (PA), hypertension, diabetes mellitus (DM), atherosclerotic cardiovascular disease (ASCVD), cancer, steroid use, estrogen use, serum uric acid, serum albumin, estimated glomerular filtration rate (eGFR), serum calcium, serum phosphorus, and total 25-hydroxyvitamin D.

*TC* Total cholesterol, *HDL-C* High-density lipoprotein cholesterol, *OR* Odds ratio, *CI* Confidence interval.

### Nonlinear dose-response relationship between TC/HDL-C and osteoporosis risk

**Fig. 2:**
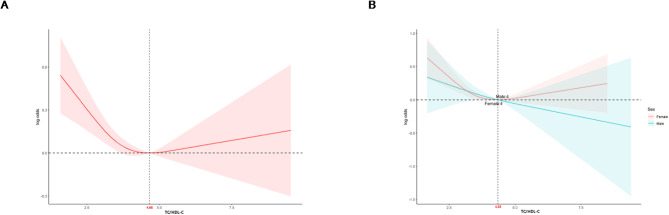
(**A**) RCS analysis of the association between TC/HDL-C ratio and osteoporosis in all participants. (**B**) RCS analysis of the association between TC/HDL-C ratio and osteoporosis in female (red) and male (blue) participants. Adjusted for covariates in model 3. *RCS* Restricted cubic spline, *TC* Total cholesterol, *HDL-C* High-density lipoprotein cholesterol.

We investigated the dose-response relationship between TC/HDL-C and osteoporosis prevalence using logistic regression with a restricted cubic spline model. A significant U-shaped association was observed between TC/HDL-C and osteoporosis (inflection point: 4.66, nonlinear *p* = 0.005; Fig. 2A). Sex-specific analysis revealed a linear positive association in males (nonlinear *p* = 0.7923), while a U-shaped relationship was found in females (inflection point: 4.35, nonlinear *p* = 0.0016; Fig. 2B). For TC/HDL-C levels below the threshold, each one-unit increase in TC/HDL-C was associated with a 17% decrease in the odds of osteoporosis (OR: 0.83, 95% CI: 0.72–0.94) in all participants and a 21% decrease (OR: 0.79, 95% CI: 0.67–0.94) in females (Table 3). However, no significant association was observed for TC/HDL-C levels above the threshold in either group (*p* > 0.05).

**Table 3:** Threshold effect analysis of TC/HDL-C ratio and osteoporosis.

Group	OR (95% CI)	*p*-value
Osteoporosis for all participants		
Inflection point (TC/HDL-C)	4.66	
TC/HDL-C < 4.66	0.83(0.72,0.94)	0.010
TC/HDL-C ≥ 4.66	0.92(0.77, 1.11)	0.400
Osteoporosis for female participants		
Inflection point (TC/HDL-C)	4.35	
TC/HDL-C < 4.35	0.79(0.67,0.94)	0.010
TC/HDL-C ≥ 4.35	1.01(0.83, 1.24)	0.920

The logistic regression model was used to estimate OR and 95% CI.

Adjusted for age, sex, race, body mass index (BMI), education level, marital status, ratio of family income to poverty (PIR), smoking status, alcohol use, physical activity (PA), hypertension, diabetes mellitus (DM), atherosclerotic cardiovascular disease (ASCVD), cancer, steroid use, estrogen use, serum uric acid, serum albumin, estimated glomerular filtration rate (eGFR), serum calcium, serum phosphorus, and total 25-hydroxyvitamin D.

*TC* Total cholesterol, *HDL-C* High-density lipoprotein cholesterol, *OR* Odds ratio, *CI* Confidence interval.

### Sensitivity analysis

A sensitivity analysis was conducted by excluding individuals on lipid-lowering medications to assess the robustness of the associations between TC, HDL-C, TC/HDL-C ratio, and osteoporosis prevalence (Table 4). In the fully adjusted model (Model 3), each 10 mg/dL increase in TC was associated with a 5% reduction in the odds of osteoporosis (OR: 0.95, 95% CI: 0.92–0.98, *p* = 0.002). HDL-C showed no significant association with osteoporosis (OR: 1.04, 95% CI: 0.97–1.11, *p* = 0.25). For the TC/HDL-C ratio, each 1-unit increase was associated with an 11% reduction in the odds of osteoporosis (OR: 0.89, 95% CI: 0.82–0.98, *p* = 0.02). When categorized into quartiles, the highest quartile (Q4) was significantly associated with lower odds of osteoporosis compared to the lowest quartile (Q1) (OR: 0.71, 95% CI: 0.52–0.97, *p* = 0.03), with a statistically significant trend across quartiles (*p* for trend = 0.014). These findings confirm the robustness of the observed associations in the main analysis.

**Table 4:** Sensitivity analysis: weighted logistic regression of TC, HDL-C, and TC/HDL-C ratio in relation to osteoporosis prevalence after excluding individuals on lipid-lowering medications.

Characteristic	Model 1		Model 2		Model 3	
	OR (95%CI)	*p*	OR (95%CI)	*p*	OR (95%CI)	*p*
TC/10	1.00(0.98,1.03)	0.89	0.95(0.92,0.98)	0.001	0.95(0.92,0.98)	0.002
HDL-C/10	1.22(1.15,1.29)	< 0.0001	1.07(1.00,1.15)	0.04	1.04(0.97,1.11)	0.25
TC/HDL-C	0.79(0.73,0.85)	< 0.0001	0.86(0.79,0.95)	0.002	0.89(0.82,0.98)	0.02
Q1	Reference	–	Reference	–	Reference	–
Q2	0.76(0.57,1.00)	0.05	0.75(0.56,1.00)	0.05	0.83(0.62,1.11)	0.21
Q3	0.53(0.39,0.71)	< 0.0001	0.62(0.45,0.85)	0.004	0.72(0.52,1.00)	0.05
Q4	0.45(0.35,0.59)	< 0.0001	0.61(0.45,0.82)	0.001	0.71(0.52,0.97)	0.03
*p* for trend		< 0.0001		< 0.001		0.014

Model 1: Unadjusted.

Model 2: Adjusted for age, sex, and race.

Model 3: Adjusted for age, sex, race, body mass index (BMI), education level, marital status, ratio of family income to poverty (PIR), smoking status, alcohol use, physical activity (PA), hypertension, diabetes mellitus (DM), atherosclerotic cardiovascular disease (ASCVD), cancer, steroid use, estrogen use, serum uric acid, serum albumin, estimated glomerular filtration rate (eGFR), serum calcium, serum phosphorus, and total 25-hydroxyvitamin D.

*TC* Total cholesterol, *HDL-C* High-density lipoprotein cholesterol, *OR* Odds ratio, *CI* Confidence interval.

### Stratified analyses

We performed subgroup analyses to further assess the relationship between TC/HDL-C and osteoporosis (Table 5). The effect varied significantly with age (*p*-interaction = 0.016). In participants aged ≥ 70 years, higher TC/HDL-C was associated with a reduced risk of osteoporosis (OR: 0.864, 95% CI: 0.789–0.946, *p* = 0.002), while no significant association was found in those aged < 70 years (*p* = 0.885). Racial differences were also observed, with non-Hispanic Whites showing a significant correlation (OR: 0.914, 95% CI: 0.842–0.992, *p* = 0.032), but no significant effects were detected in other ethnic groups. Although BMI, eGFR, and calcium showed no interactions, higher TC/HDL-C was linked to a reduced risk of osteoporosis in participants with phosphorus levels < 1.2 mmol/L (OR: 0.882, 95% CI: 0.783–0.994, *p* = 0.040) and total 25-hydroxyvitamin D levels ≥ 70 nmol/L (OR: 0.887, 95% CI: 0.806–0.976, *p* = 0.015).

**Table 5:** Subgroup logistic regression analysis of the association between TC/HDL-C ratio and osteoporosis in elderly individuals.

Characteristic	OR (95%CI)	*p*	*p* for interaction
Age, years			0.016
>= 70	0.864(0.789,0.946)	0.002	
< 70	0.992(0.892,1.104)	0.885	
Sex			0.875
Female	0.922(0.848,1.003)	0.059	
Male	0.901(0.785,1.035)	0.137	
Race			0.847
Mexican American	0.846(0.708, 1.010)	0.063	
Non-Hispanic Black	0.966(0.814, 1.147)	0.688	
Non-Hispanic White	0.914(0.842,0.992)	0.032	
Other Race	0.966(0.796,1.173)	0.720	
Body mass index, kg/m^2^			0.309
< 30	0.931(0.860,1.007)	0.073	
>= 30	0.995(0.873, 1.135)	0.942	
eGFR, mL/min/1.73 m^2^			0.916
< 60	0.879(0.764,1.011)	0.071	
>= 60	0.935(0.864,1.012)	0.093	
Calcium, mmol/L			0.941
< 2.35	0.920(0.835,1.014)	0.092	
>= 2.35	0.916(0.814, 1.030)	0.139	
Phosphorus, mmol/L			0.485
< 1.2	0.882(0.783,0.994)	0.040	
>= 1.2	0.954(0.873,1.042)	0.286	
Total 25-hydroxyvitamin D, nmol/L			0.09
< 70	0.973(0.883,1.071)	0.565	
>= 70	0.887(0.806,0.976)	0.015	

*TC* Total cholesterol, *HDL-C* High-density lipoprotein cholesterol, *OR* Odds ratio, *CI* Confidence interval.

## Discussion

This study investigates the relationship between the TC/HDL-C ratio and osteoporosis in older adults using NHANES data from 2005 to 2020. Our findings indicate an inverse association between a higher TC/HDL-C ratio and the risk of osteoporosis, which remained significant even after adjusting for multiple covariates. Notably, a U-shaped relationship was observed, particularly among women, suggesting that the protective effects of a higher TC/HDL-C ratio may plateau beyond a certain threshold. Stratified analyses revealed that this association was more pronounced in adults aged 70 or older, non-Hispanic Whites, and individuals with specific levels of phosphorus and 25-hydroxyvitamin D.

Existing evidence suggests that lipid metabolism plays an important role in bone health, particularly in the development of osteoporosis. Several studies have reported an inverse relationship between lipid profiles and bone mineral density. For example, Kim et al. found a significant negative correlation between the TC/HDL-C ratio and bone mineral density in South Korean men, supporting our findings of a protective effect of higher TC/HDL-C ratios against osteoporosis^9^. The study’s focus on men, who generally have higher bone density and different hormonal profiles than women^25–27^, may partially explain the observed inverse association, as the protective effects of lipid metabolism might be more apparent in this group. In contrast, Alissa et al. examined postmenopausal women with coronary artery disease, a group with distinct clinical characteristics including advanced age, estrogen deficiency, and pre-existing cardiovascular conditions^28^. These factors not only increase the risk of osteoporosis but also amplify the potential impact of elevated TC/HDL-C ratios on bone metabolism. The inclusion of women with coronary artery disease likely heightened the observed inverse association between atherogenic indices, including the TC/HDL-C ratio, and osteoporosis risk, as lipid-related oxidative stress and inflammation are more pronounced in this population^29,30^. These studies reinforce the notion that lipid metabolism may be linked to bone health through mechanisms yet to be fully elucidated. However, not all studies align with this view. For instance, Go et al. observed no significant relationship between cholesterol levels and bone mineral density in postmenopausal Korean women, contrasting with the inverse associations found in our study^11^. Our findings further suggest that in individuals aged 60 to 69 years, the association between the TC/HDL-C ratio and osteoporosis was not significant (*p* = 0.885). Similar to younger populations (< 60 years), where lipid metabolism appears to play a less prominent role in bone health, other factors such as physical activity, diet, and metabolic health may exert greater influence^12^. Furthermore, the lower prevalence of osteoporosis in these groups reduces statistical power to detect meaningful associations. Future studies are needed to elucidate these variations and the underlying mechanisms linking lipid profiles to bone health across different populations.

Cholesterol, particularly the balance between TC and HDL-C, plays a crucial role in bone metabolism. Cholesterol is a precursor for steroid hormones, such as estrogen, which are vital for maintaining bone density, especially in postmenopausal women^8^. A higher TC/HDL-C ratio, indicating higher total cholesterol or lower HDL-C, could disrupt this balance, negatively affecting bone turnover. HDL-C, often regarded as the “good cholesterol,” has anti-inflammatory and antioxidative effects, protecting osteoblasts (bone-forming cells) from oxidative stress, thereby promoting bone formation^5^. Conversely, lower levels of HDL-C or elevated TC/HDL-C ratios might contribute to increased osteoclast (bone-resorbing cells) activity, accelerating bone loss^9^. Several studies have shown an inverse association between TC/HDL-C ratios and bone mineral density, suggesting a protective role when the ratio is balanced^31^. However, the present study found an inverse relationship between the TC/HDL-C ratio and osteoporosis, indicating that higher ratios were associated with lower osteoporosis risk. This paradox may be explained by the combined effects of cholesterol and HDL-C on bone metabolism. Cholesterol serves as a precursor for steroid hormones such as estrogen and testosterone, which are critical for maintaining bone density, particularly in aging populations^32^. In contrast, elevated HDL-C levels, while protective in cardiovascular health, may under certain conditions impair bone remodeling by suppressing osteoblast activity or promoting bone resorption^33^. The TC/HDL-C ratio reflects the balance between these opposing effects, with a higher ratio indicating reduced HDL-C dominance, potentially allowing the protective effects of cholesterol-derived hormones to prevail. Additionally, metabolic and inflammatory contexts, such as reduced oxidative stress at higher TC/HDL-C ratios, may further support bone health^34,35^. This interplay highlights the complex role of lipid metabolism in bone health.

The relationship between lipids and bone health appears to differ between men and women. In females, a U-shaped association between the TC/HDL-C ratio and osteoporosis was observed, where the risk decreases as the ratio rises, up to a threshold, beyond which the protective effect stabilizes^36^. This trend may be attributed to the interaction between lipids and estrogen, which is crucial for maintaining bone mass. In postmenopausal women, HDL-C is positively associated with bone mineral density. Therefore, it can be inferred that when the TC/HDL-C ratio is elevated, postmenopausal women are more susceptible to osteoporosis^11^. In males, the relationship is more linear, likely due to more stable levels of testosterone and its relatively weaker interactions with lipid metabolism compared to estrogen^37^. This suggests that the effects of lipid levels on bone health are more pronounced in females, particularly during and after menopause.

This study has several strengths, including the use of a large, representative sample from NHANES, enhancing the generalizability of the findings to the U.S. population. Comprehensive adjustments for confounders such as demographic, lifestyle, and clinical variables help ensure the robustness of the results. Additionally, advanced statistical techniques like restricted cubic spline models and interaction analyses were employed to capture nonlinear relationships and explore subgroup differences. However,

This study has several limitations that should be acknowledged. First, the cross-sectional design precludes any causal inferences between the TC/HDL-C ratio and osteoporosis risk. Second, variability in lipid measurement techniques and potential recall bias in self-reported osteoporosis diagnoses may affect data accuracy. Third, while efforts were made to adjust for multiple confounders, the possibility of residual confounding remains, as some unmeasured factors (e.g., physical activity levels, dietary calcium intake) could still influence the observed associations. Fourth, the study population was limited to adults aged 60 years and above, which precludes generalizability to younger populations. Age-related physiological changes in lipid metabolism and bone remodeling may differentially influence the TC/HDL-C ratio and osteoporosis risk in younger individuals. Finally, although we utilized a nationally representative sample, potential selection bias should be considered. Participants in NHANES may systematically differ from non-participants in terms of health-seeking behaviors or socioeconomic status, which could affect the external validity of our findings. Future longitudinal studies with broader age inclusion and more comprehensive covariate assessments are needed to validate these results.

Lipid ratios, particularly the TC/HDL-C ratio, show promise as biomarkers for assessing osteoporosis risk, especially in older adults. Given the observed sex differences, there is a need for gender-specific guidelines in managing cholesterol and osteoporosis risk. This is particularly crucial for individuals over 70, where the association between lipid profiles and bone health is most pronounced. Future research should focus on longitudinal studies to establish causality between cholesterol levels and bone density. Interventional trials are needed to explore whether modifying the TC/HDL-C ratio can help prevent or mitigate osteoporosis. Additionally, mechanistic studies are necessary to uncover the biological pathways linking lipid metabolism to bone health, with particular attention to the role of sex hormones. Moreover, collaborative efforts are needed to establish standardized protocols for future meta-analyses in this emerging field, which will help to synthesize the heterogeneous evidence and provide more comprehensive insights into the relationship between lipid profiles and osteoporosis risk.

## Conclusions

This study demonstrated a significant inverse association between the TC/HDL-C ratio and osteoporosis in older adults, with a U-shaped relationship observed in females. The findings suggest that incorporating TC/HDL-C ratio assessments could enhance the screening process for osteoporosis risk, particularly in the elderly population. Monitoring this lipid ratio may serve as a practical target for early intervention, potentially reducing the risk of osteoporosis and related fractures.

## Electronic supplementary material

Below is the link to the electronic supplementary material.

Supplementary Material 1

Supplementary Material 2

## Data Availability

The NHANES data analyzed in this study can be accessed via the following link: https://www.cdc.gov/nchs/nhanes.
