# Supplementary material for: U-shaped association between TC/HDL-C ratio and osteoporosis risk in older adults
Source: Sci Rep. 2025 Feb 8;15:4791. doi: 10.1038/s41598-025-89537-5 (PMC11807207; doi:10.1038/s41598-025-89537-5)
Supplement: Supplementary file 1 — Supplementary Material 1 [file 41598_2025_89537_MOESM1_ESM.pdf]

SPRINGER NATURE

Change of authorship request form - Journals (pre-acceptance)

Important information. Please read.

- This form should be used by authors to request any change in authorship (adding/deleting authors) including changes in corresponding authors. This form should not be used for name changes. Please fully complete all sections. Use black ink and block capitals and provide each author's full name with the given name first followed by the family name.

- By signing this declaration, all authors guarantee that the order of the authors are in accordance with their scientific contribution, if applicable as different conventions apply per discipline, and that only authors have been added who made a meaningful contribution to the work.

- Please note, in author collaborations where there is formal agreement for representing the collaboration, it is sufficient for the representative or legal guarantor (usually the corresponding author) to complete and sign the Authorship Change Form on behalf of all authors, **next to the added/removed author(s)**. **(Complete Section 3, followed by Section 6.)**
  In author collaborations where there is no formal agreement for representing the collaboration and **there are more than 10 authors**, one may sign for all, provided the signer appends correspondence that attests that each of the authors have agreed to the change **and the added/removed authors sign the form**. **(Complete Section 3, followed by Section 6.)**

- Please note, we cannot investigate or mediate any authorship disputes. If you are unable to obtain agreement from all authors (including those who you wish to be removed) you must refer the matter to your institution(s) for investigation. Please inform us if you need to do this.

- If you are not able to return a fully completed form within **30 days** of the date that it was sent to the author requesting the change, we may have to withdraw your manuscript. We cannot publish manuscripts where authorship has not been agreed by all authors (including those who have been removed).

- Incomplete forms will be rejected.

- Please return/upload this form, fully completed, to the Journals Editorial Office. The Journal and/or Publisher will consider the information you have provided to decide whether to approve the proposed change in authorship. We may decide to contact your institution for more information or undertake a further investigation, if appropriate, before making a final decision.

SPRINGER NATURE Springer Nature is one of the world's leading global research, educational and professional publishers, created in May 2015 through the combination of Nature Publishing Group, Palgrave Macmillan, Macmillan Education and Springer Science+Business Media.

SPRINGER NATURE

Change of authorship request form - Journals (pre-acceptance)

Section 1: Please provide the current title of manuscript

Manuscript ID no.: 494494bd-2a9c-4a83-bcaf-0e60885d8857

Title: U-shaped association between total cholesterol to high-density lipoprotein cholesterol ratio and osteoporosis risk in older adults

Section 2: Please provide the previous authorship, in the order shown on the manuscript before the changes were introduced. Please indicate the corresponding author by adding (CA) behind the name.

	First name(s)	Family name	ORCID or SCOPUS id, if available
1st author	Chuanwei	Zhao(CA)	0009-0000-0548-7573
2nd author	Haijie	Yang(CA)	
3rd author	Yane	Yang	
4th author	Mu	Lin	
5th author	Wenli	Qiao	
6th author	Zeyao	Hu	
7th author			
8th author			
9th author			
10th author			

Please use an additional sheet if there are more than 10 authors.

SPRINGER NATURE Springer Nature is one of the world's leading global research, educational and professional publishers, created in May 2015 through the combination of Nature Publishing Group, Palgrave Macmillan, Macmillan Education and Springer Science+Business Media.

SPRINGER NATURE

Change of authorship request form - Journals (pre-acceptance)

Section 3: Please provide a justification for change. Please use this section to explain your reasons for changing the authorship of your manuscript, e.g. what necessitated the change in authorship? Please refer to the (journal) policy pages for more information about authorship. Please explain why omitted authors were not originally included and/or why authors were removed on the submitted manuscript.

The changes in authorship are necessary due to the following reasons:
Addition of Authors:After further consideration and review, it was determined that additional individuals made significant contributions to the manuscript that warrant authorship. These contributions include substantial involvement in the research design, data analysis, and manuscript writing.
Adjustment of Author Order:The order of authors has been adjusted to more accurately reflect the relative contributions of each author to the manuscript. This adjustment ensures that the authorship order is in accordance with the conventions of our field and the guidelines of the journal.
Originally, some contributors were not included as authors because their contributions were not fully recognized at the time of initial submission. However, upon further evaluation, it became clear that their involvement meets the criteria for authorship. The changes have been discussed and agreed upon by all authors, including those being added.

Section 4: Proposed new authorship. Please provide your new authorship list in the order you would like it to appear on the manuscript. Please indicate the corresponding author by adding (CA) behind the name. <u>If the Corresponding Author has changed, please indicate the reason under section 3</u>.

	First name(s)	Family name (this name will appear in full on the final publication and will be searchable in various abstract and indexing databases)	Affiliated institute	E-mail address
1st author	Chuanwei	Zhao(CA)	The Second People's Hospital of Baosha	zhaochuanwei946@163.com
2nd author	Xiaochun	Zhang	The Second People's Hospital of Baosha	949692318@qq.com
3rd author	Xu	Zhang	The Second People's Hospital of Baosha	3140705506@qq.com
4th author	Bo	Zhao	The Second People's Hospital of Baosha	1565804716@qq.com
5th author	Yane	Yang	The Second People's Hospital of Baosha	2389081196@qq.com
6th author	Mu	Lin	The Second People's Hospital of Baosha	546699462@qq.com
7th author	Wenli	Qiao	The Second People's Hospital of Baosha	qiaoqiao20202020@126.com
8th author	Zeyao	Hu	The Second People's Hospital of Baosha	1938094811@qq.com
9th author	Haijie	Yang(CA)	The Second People's Hospital of Baosha	phdyang@aliyun.com
10th author				

Please use an additional sheet if there are more than 10 authors.

SPRINGER NATURE Springer Nature is one of the world's leading global research, educational and professional publishers, created in May 2015 through the combination of Nature Publishing Group, Palgrave Macmillan, Macmillan Education and Springer Science+Business Media.

Change of authorship request form - Journals (pre-acceptance)

Section 5: Author contribution, Acknowledgement and Disclosures. Please use this section to provide a new disclosure statement and, if appropriate, acknowledge any contributors who have been removed as authors and ensure you state what contribution any new authors made (if applicable per the journal or book (series) policy). Please ensure these are updated in your manuscript - after approval of the change(s) - as our production department will not transfer the information in this form to your manuscript.

New acknowledgements:

Not applicable

New Disclosures (financial and non-financial interests, funding):

Not applicable

New Author Contributions statement (if applicable per the journal policy):

C.Z., X.C.Z., X.Z., and B.Z. were pivotal in conceptualizing the research and overseeing the design. Y.Y., M.L., W.Q., and Z.H. spearheaded the data analysis and interpretation. Y.Y., M.L., and Z.H. took on the responsibility of creating the figures and tables. W.Q. and H.Y. drafted the initial version of the manuscript. C.Z. and H.Y., as the corresponding authors, ensured the integrity and accuracy of the work.

State 'Not applicable' if there are no new authors.

SPRINGER NATURE Springer Nature is one of the world's leading global research, educational and professional publishers, created in May 2015 through the combination of Nature Publishing Group, Palgrave Macmillan, Macmillan Education and Springer Science+Business Media.

SPRINGER NATURE

Change of authorship request form - Journals (pre-acceptance)

Section 6: Declaration of agreement. *All* authors, unchanged, new and removed *must* sign this declaration.
(NB: Please print the form, (docu)-sign and return/upload a scanned copy. Please note that signatures that have been inserted as an image file are acceptable as long as it is handwritten. Typed names in the signature box are unacceptable.) *** Please delete as appropriate. Delete all of the bold if you were on the original authorship list and are remaining as an author.**

	First name	Family name		Signature	Date
1st author	Chuanwei	Zhao	I agree to the proposed new authorship shown in section 4 /**and the addition**/and the proposed change in corresponding author	Chuanwei zhao	January 7, 2025
2nd author	Xiaochun	Zhang	I agree to the proposed new authorship shown in section 4 /**and the addition**/and the proposed change in corresponding author	Xiaochun Zhang	January 7, 2025
3rd author	Xu	Zhang	I agree to the proposed new authorship shown in section 4 /**and the addition**/and the proposed change in corresponding author	Xu zhang	January 7, 2025
4th authors	Bo	Zhao	I agree to the proposed new authorship shown in section 4 /**and the addition**/and the proposed change in corresponding author	Bo zhao	January 7, 2025
5th author	Yane	Yang	I agree to the proposed new authorship shown in section 4 /**and the addition**/and the proposed change in corresponding author	Yane Yang	January 7, 2025
6th author	Mu	Lin	I agree to the proposed new authorship shown in section 4 /**and the addition**/and the proposed change in corresponding author	Mu Lin	January 7, 2025
7th author	Wenli	Qiao	I agree to the proposed new authorship shown in section 4 /**and the addition**/and the proposed change in corresponding author	Wenli Qiao	January 7, 2025

SPRINGER NATURE Springer Nature is one of the world's leading global research, educational and professional publishers, created in May 2015 through the combination of Nature Publishing Group, Palgrave Macmillan, Macmillan Education and Springer Science+Business Media.

Change of authorship request form - Journals (pre-acceptance)

	First name	Family name		Signature	Date
8th author	Zeyao	Hu	I agree to the proposed new authorship shown in section 4 **/and the addition/**and the proposed change in corresponding author	Zeyao Hu	January 7, 2025
9th author	Haijie	Yang	I agree to the proposed new authorship shown in section 4 **/and the addition/**and the proposed change in corresponding author	Haijie Yang	January 7, 2025
10th author			I agree to the proposed new authorship shown in section 4 **/and the addition/**and the proposed change in corresponding author		

Please use an additional sheet if there are more than 10 authors.

In case of author collaborations with formal agreement:

	Name of consortium/consortia	First name	Family name		Signature	Date
Representative/legal guarantor				I agree to the proposed new authorship shown in section 4 **/and the addition/removal*of my name to the authorship list** /and the proposed change in corresponding author		

Both added/removed authors should complete the information in the first table under Section 6.

---- End of form ----

SPRINGER NATURE Springer Nature is one of the world's leading global research, educational and professional publishers, created in May 2015 through the combination of Nature Publishing Group, Palgrave Macmillan, Macmillan Education and Springer Science+Business Media.
