# Supplementary material for: U-shaped association between TC/HDL-C ratio and osteoporosis risk in older adults
Source: Sci Rep. 2025 Feb 8;15:4791. doi: 10.1038/s41598-025-89537-5 (PMC11807207; doi:10.1038/s41598-025-89537-5)
Supplement: Supplementary file 2 — Supplementary Material 2 [file 41598_2025_89537_MOESM2_ESM.docx]

# List of abbreviations

AIC: Akaike Information Criterion

ASCVD: Atherosclerotic Cardiovascular Disease

BMD: Bone Mineral Density

BMI: Body Mass Index

CDC: Centers for Disease Control and Prevention

CI: Confidence Interval

CKD-EPI: Chronic Kidney Disease Epidemiology Collaboration

DXA: Dual-Energy X-Ray Absorptiometry

eGFR: Estimated Glomerular Filtration Rate

GED: General Educational Development

HDL-C: High-Density Lipoprotein Cholesterol

LDL-C: Low-Density Lipoprotein Cholesterol

MET: Metabolic Equivalent of Task

NHANES: National Health and Nutrition Examination Survey

OR: Odds Ratio

PIR: Poverty Income Ratio

RCS: Restricted Cubic Spline

TC: Total Cholesterol

TG: Triglycerides

WHO: World Health Organization
